# Effects of Ability and Effort Praise on Children’s Failure Attribution, Self-Handicapping, and Performance

**DOI:** 10.3389/fpsyg.2018.01883

**Published:** 2018-10-02

**Authors:** Shufen Xing, Xin Gao, Ying Jiang, Marc Archer, Xia Liu

**Affiliations:** ^1^Department of Psychology, Capital Normal University, Beijing, China; ^2^Institute of Developmental Psychology, Beijing Normal University, Beijing, China; ^3^School of Public Health, Zhejiang University, Hangzhou, China

**Keywords:** praise, ability, effort, failure attribution, self-handicapping

## Abstract

Previous research has suggested that children praised for ability are more likely to attribute their failure to low ability compared to those who are praised for effort. At the same time, self-worth theory suggests that when an individual’s self-worth is threatened, they are likely to use a self-serving attributional strategy and self-handicapping. From the perspective of self-worth theory, the present study investigated how ability and effort praise influenced children’s failure attribution, self-handicapping, and their subsequent performance compared to simple informational feedback. Fifth graders (*N* = 103, average age = 11.2 years, *SD* = 0.71) were randomly assigned to three praise conditions (ability, effort, or no praise). The results revealed that children praised for ability were more likely to attribute their subsequent failure to non-ability factors and indicate more claimed and behavioral self-handicapping than children who were praised for effort or not praised at all. As behavioral self-handicapping created actual obstacles to progress, children praised for ability made significantly less improvement in their performance than those in the other two groups. In addition, the findings showed that children praised for effort also adopted the claimed self-handicapping and defensive attributional strategies compared to those in the no-praise conditions. These results indicate that parents and teachers should not haphazardly administer praise. Implications for parents, teachers, and future research directions, including the replication of this study in diverse cultural settings, conditions of effort praise, and effects of other types of praise, are discussed.

## Introduction

Ability praise is a common way to provide feedback on good performance that can boost children’s sense of efficacy and motivate their learning (Koestner et al., 1989). However, the studies with these findings only focused on the effects of ability praise during an experience of success, a growing body of research has revealed that ability praise can often be ineffective after failure compared with effort praise. For example, ability praise may lead children to display a helpless response after failure, including more negative self-cognitions and affect, less persistence, and impaired performance, while effort praise leads children to focus on the process of work and development of learning skills, leading to greater persistence and good performance after setbacks (Mueller and Dweck, 1998; Gunderson et al., 2017); it also leads to better performance among undergraduates (Lessard et al., 2015). In addition, ability praise tends to put children in a fixed mind-set (ability is fixed, and you just have it), whereas effort praise tends to put them in a growth mind-set (you can develop these skills because you’re working hard; Mueller and Dweck, 1998; Gunderson et al., 2013, 2017; Haimovitz and Dweck, 2017). Research has also shown that ability praise can promote young children’s cheating behaviors (Zhao et al., 2017). In natural settings, adults may use inconsistent praise, and children reduce their persistence when hearing even a small amount of ability praise, whereas children’s self-evaluation is preserved when hearing a small amount of effort praise (Zentall and Morris, 2010).

The majority of the studies outlined above were conducted in the European–American culture; these findings may not generalize to other cultures. For example, an observational study investigated parental praise in Chinese-immigrant and European–American families found that parental praise has different functions across two cultural settings (Wang et al., 2008). Accordingly, an important extension of this line of research on the effects of praise for ability and effort on a child’s development is the consideration of cultural specificity (Henderlong and Lepper, 2002). It is possible that ability and effort praise have different effects on children’s development; indeed, it has been found that Westerners emphasize the importance of ability, while Chinese are more inclined to believe achievement centers primarily on effort (Lewis, 1995; Salili, 1996). Hence, we were interested in exploring the question of how children praised for ability or effort under conditions of success would respond to a specific failure within the cultural context of Mainland China.

In human nature, there is a need to build belief in one’s self-worth, which was referred to the individual’s sense of inherent value and the degree to which he or she accepts him or herself. Failure was considered as a threat on one’s ability and self-worth (Covington, 1992; Ferradás et al., 2016). Self-worth theory suggested that individuals would strive to give their lives meaning by pursuing the approval of others (Covington, 1992, 2009). The basic prerequisite of self-worth theory could be traced back to the notion of psychological motives such as the needs for approval and achievement which were proposed by John Atkinson’s need achievement theory. Atkinson (1957) pointed out that the inclination to achieve was the result of an emotional conflict between endeavoring success and avoiding failure. Self-worth theory basically adopted the concept of emotional conflict and assumed that individuals would devote a life-long strive to establish and maintain a sense of personal value (Covington, 1992, 2009). Thus, individuals would adopt strategies, such as self-serving failure attributions (Bodroža and Mirkov, 2011) or self-handicapping strategies (Cano et al., 2018) to avoid failure or to change its negative emotional consequences and implication (Covington, 1992; De Castella et al., 2013). Further research is needed to better understand which children are more inclined to use self-serving failure attributions or self-handicapping as a means of protecting their sense of self-worth, and under which circumstances this is more likely to occur. Berglas (1990) argued that one of the dominant factors may be parents’ or teachers’ inappropriate use of positive evaluative feedback—praise, compliments, and the like. Thus, ability or effort praise as a kind of environmental cue may influence children’s use of self-serving failure attributions or self-handicapping strategies. From the perspective of self-worth theory, the main objectives of present study were to examine two research questions. First, would ability praise lead children to use more defensive attributional or self-handicapping strategies when they face the threat of subsequent failure compared to effort praise and simple informational feedback? Second, is effort praise always beneficial to children? Would praise for effort also lead children to use more self-serving failure attributions or self-handicapping strategies compared to simple informational feedback?

### Failure Attribution: Blame Ability or Preserve Self-Worth?

According to attribution theory, individuals tend to identify causes of achievement outcomes; particularly in instances of failure, achievement is generally understood to depend upon ability and effort (Weiner, 1994). Therefore, praise for ability or effort—which provides potential information on causation—may affect children’s attributions when they experience failure. Mueller and Dweck (1998) launched a series of experimental studies to examine the effects of ability and effort praise on children’s failure attribution. Experimenters let fifth graders work on a set of Raven’s progressive matrices of moderate difficulty, and then praised their good performance. Some children received ability praise (*You must be smart at these problems)*, some received effort praise *(You must have worked hard at these problems)*, while the remaining in the control condition were given non-directed positive feedback. *(Wow, you did very well on these problems. You got [number of problems] right. That’s a really high score*.*)* Then, all of the children were given a more difficult set of 10 progressive matrices, after which the experimenters told them that they had performed poorly. Finally, children were asked to describe why they thought they had not done well. The findings found that children praised for ability during a successful event were more likely to attribute their subsequent failure to low ability compared to those praised for effort and in the control, and they were the least likely among the three groups to attribute their failure to low effort. In discussing their findings, Mueller and Dweck (1998) inferred that children who experience praise of ability after successes tend to generalize these experiences to an understanding that ability underpins performance; subsequent failure in more challenging tasks is also attributed to ability. However, children praised for ability attribute subsequent failure to their lower self-ability, which runs contrary to the basic premise of self-worth theory, which posits that people are motivated to establish and maintain a sense of self-worth, individuals are less afraid of failure itself than of the failure being attributed to their low ability, threats to self-worth often give rise to defensive coping strategies, such as biased self-serving attributional strategies (Covington, 1992, 2009).

On account of self-worth theory, we hypothesized that compared to those who were praised for effort and only received simple informational feedback during a successful performance, children praised for ability would use more defensive attribution and contribute their subsequent failure to non-ability factors (e.g., test anxiety or lack of test time) to protect their self-worth.

### Self-Handicapping: Reduce the Pain of Failure

Self-handicapping, also considered a defensive self-protection strategy, refers to the various ways in which people create obstacles for themselves to provide an *a priori* excuse for possible failure in the future in order to ensure that inability is not blamed and self-worth is preserved (Snyder et al., 2014; Clarke and Maccann, 2016). In the event of success despite self-handicapping, self-perceptions of ability are elevated and feelings of self-worth are improved or maintained (Berglas and Jones, 1978; Covington, 1992, 2009). Generally speaking, two forms of self-handicapping strategies have been identified: behavioral and claimed (Schwinger et al., 2014). In cases of behavioral self-handicapping, an actual obstacle that directly impedes performance is created. For example, a child may give up learning or reduce their efforts before a test (Leary and Shepperd, 1986). In contrast, claimed self-handicapping involves only reporting the presence of obstacles, for example, a child may work hard for a test but tell her schoolmates that she hardly prepared, or claim to suffer from test anxiety (Hirt et al., 1991) or a bad mood (Baumgardner et al., 1985), suggested that threats to self-worth can lead individuals to engage in self-worth protection through the use of self-handicapping (Covington, 1992, 2009). For example, it has been found that gifted children—who have most likely experienced repeated confirmation of their exceptional abilities—are more inclined to use self-handicapping behaviors when there is cause for uncertainty concerning future success (Snyder et al., 2014). It is not surprising that children who received praise for ability after successes would feel threatened after they experienced a setback in their achievement. Because failure is usually regarded as a signal of low ability and associated with low level of self-worth. Therefore, they are more inclined to use self-handicapping strategies to protect their self-worth. In the current study, we adopted the self-reported level of test anxiety as the indicator of claimed self-handicapping, the time of completing post-failure task as the indicator of behavioral self-handicapping, and explored whether praise for ability would make children use more self-handicapping strategies when they were faced with the threat of subsequent failure.

Based on self-worth theory, we hypothesized that compared to those who received effort praise and simple informational feedback during a successful task, children praised for ability would be more likely to capitalize on claimed self-handicapping strategies (i.e., report higher levels of test anxiety) and behavioral self-handicapping strategies (i.e., use less time of completing post-failure task), and thus show worse performance after failure.

### Effort Praise: Is Always Beneficial?

Much evidence has been shown for the positive effects of effort praise (Mueller and Dweck, 1998; Zentall and Morris, 2010; Gunderson et al., 2013, 2017). However, the benefits of effort praise may be limited if hard work results in setback or if effort is overemphasized, and simple informational feedback with no praise component might be the best response in case of hard work resulting in failure (Henderlong and Lepper, 2002). Unfortunately, the research paradigm of Mueller and Dweck (1998) did not allow us to test this possibility as children in the control also received initial positive feedback statements. Consequently, it is extraordinarily essential to launch a study with the inclusion of a no-praise control group, who only receive simple informational feedback, to examine the absolute effects of effort praise on child’s responses. How, then, might children whose effort has been praised after good performance make their attributions for subsequent failures compared to those who received simple informational feedback during a successful task? Studies from Nicholls (1978, 1984) found that younger children do not distinguish ability and effort as separate dimensions in their causal reasoning until approximately third grade, whereas older children (at ages older than 11) believe that effort and ability have a compensatory relationship which means that greater effort implies lower ability or effort and ability are related negatively. Therefore, children (at ages older than 11) who received praise for effort after successes in this study may also feel their self-worth be threatened after they experienced a setback because of the compensatory relationship between effort and ability, they might utilize biased self-serving failure attributions or self-handicapping strategies to alter the meaning of failure and then protect their sense of self-worth.

Based on self-worth theory, we hypothesized that the benefits of effort praise for older children are limited if hard work results in setback. Compared to those who only received simple informational feedback during a successful task, children praised for effort also tend to adopt defensive attributional strategies and self-handicapping strategies to protect their self-worth in subsequent failure.

## Materials and Methods

### Participants

One hundred and six fifth graders (58 boys, 48 girls) between 10 and 13 years of age (*M* = 11.19, *SD* = 0.56 for boys and *M* = 11.26, *SD* = 0.56 for girls) were recruited from two public elementary schools in Beijing. All of the participants were without learning difficulties or any other disorders. Parental written consent to participate in the research was obtained for all of the participants. Participants were randomly allocated to one of the three feedback conditions: (1) ability praise (*n* = 37): “*Wow, you did a good job, you got [number of problems] correct. That’s a really high score! I can see you must be very clever!*”; (2) effort praise (*n* = 36): “*Wow, you did a good job, you got [number of problems] correct. That’s a really high score! I can see you must have worked very hard to correctly solve these problems*”; or (3) no praise (*n* = 33): “*You got [number of problems] right.*” Regardless of the children’s actual score, the experimenter told every child that they had gotten at least 80% of the problems right. Three participants were excluded from analysis because they could solve fewer than three of the first set of problems, so a total of 103 children were included in the main analyses (37 in the ability praise condition, 35 in effort praise, and 31 in no praise).

### Measures

*Standard progressive matrices* (Raven et al., 1998) were used to establish the experimental conditions of success or failure. The children’s scores were derived from three sets of 10 problems.

*Failure attribution* was measured by providing children with a list of four factors (low ability, low effort, test anxiety, and lack of test time) to which they could attribute their failure. Children were asked to indicate how likely it was that each factor had caused their failure. Each item was rated on a six-point Likert scale (*0 = not at all likely, 5 = very likely*).

*Claimed self-handicapping* was measured by the level of text anxiety reported by children before they suffered failure. Children were asked to respond to the question “*How anxious did you feel when you finished these problems?*” Experimenters let children assess their level of text anxiety on a six-point Likert scale (*0 = not at all, 5 = very much*).

*Behavioral self-handicapping* was operationalized as the time of completing post-failure task, calculated by measuring the time taken to complete all of the problems from sets 1 to 3.

### Manipulation Check

Children responded to two items at the completion of the study: “*Was your ability important for your performance?*” and “*Was your effort important for your performance?*” Each item was rated on a six-point Likert scale (*0 = not at all important*, *5 = very important*). This manipulation check was used to test whether or not children had understood and believed the feedback the experimenter had given.

### Procedure

The study was approved by the Research Ethics Committee of Capital Normal University, and parental written consent to participate in the research was obtained for all of the participants before the experiments. Each child was tested individually. An experimenter greeted the child and guided them into an empty classroom. After a brief tutorial for solving the progressive matrices, children were asked to work on the first set of progressive matrices with medium difficulty (set 1) and also told that there was a 5-min time limit for these problems. After 5 min or upon completion of all of the problems, the experimenter ended the task, calculated the score, and then gave feedback according to the participant condition (i.e., ability praise, effort praise, or no praise). Claimed self-handicapping was then measured by asking the participant to report their level of test anxiety.

Next, the experimenter introduced the second, more difficult set of progressive matrices (set 2) and again gave the child 5 min to work on them. This time children in all three of the groups were provided with failure feedback: “*It looks like you had some trouble with these difficult problems*—*you performed poorly on them because only 2 or 3 answers are correct.*” After participants received the failure feedback, they were asked to once again make attributions for failure, as described above.

Subsequently, children were asked to work on a third, medium-difficulty set of progressive matrices (set 3) without a time limit. The time spent on the third set of problems was used as the index of behavioral self-handicapping. The number of correct answers on the third set was used as the post-failure performance.

Finally, the experimenter asked children to assess the importance of ability and effort in their performance. At the end of the experiment, the children were debriefed: The experimenter explained that the second set was beyond their current knowledge and at the level of students in junior year 1, ensuring that all of children were proud of their performance when they left the experimental classroom.

### Data Analysis

First, preliminary analysis compared children’s ratings on the importance of ability and effort among the three praise conditions to examine the validity of the experimental manipulations. It would be taken that children understood and were influenced by the experimenter’s type of praise for their performance if there were differences in children’s attitudes regarding ability and effort among the three groups. Preliminary analysis also compared whether performance and time spent working before encountering failure were equal among children in the three conditions. Then, analysis of variance was conducted to examine the differences in children’s failure attribution and self-handicapping among the three groups. Effect size is determined through partial eta squared. The effect is considered small when ranging between η*_p_*^2^ = 0.01 and η*_p_*^2^ = 0.06; medium when ranging between η*_p_*^2^ = 0.06 and η*_p_*^2^ = 0.14; and large when η*_p_*^2^ > 0.14.

## Results

### Preliminary Analysis

First, we conducted two one-way ANOVAs to examine whether children in the three conditions differed in time spent working and performance on the first set of matrices. The results showed that there was no significant difference in the time spent working on set 1 across the three conditions (see **Table 3**). Similarly, children in the three conditions did not differ in performance on the first set of problems (**Table 4**). These results indicated that children in the three conditions were equal in their performance and time spent working before they encountered failure.

To examine whether children had understood and believed the feedback given by the experimenter, one-way ANOVAs were conducted. The results showed that children’s ratings of the importance of ability differed among the three groups (see **Table 1**) with a large effect size. *Post hoc* tests indicated that children praised for ability rated ability as significantly more important than children praised for effort (mean difference = 0.98, *SE* = 0.18, *p* < 0.01) and those not praised (mean difference = 0.95, *SE* = 0.19, *p* < 0.01). There was no significant difference between children praised for effort and those not praised (mean difference = -0.04, *SE* = 0.19, *p* > 0.05).

**Table 1:** Descriptive statistics for children’s ratings on the importance of ability and effort.

		Ability group (*n* = 37)	Effort group (*n* = 35)	Control group (*n* = 31)	Total (*n* = 103)	*F*_(2,100)_	η*_p_*^2^
Importance of ability	*M*	5.27	4.29	4.32	4.63	18.24^∗∗^	0.27
	*SD*	0.13	0.13	0.14	0.08		
Importance of effort	*M*	4.16	5.29	4.87	4.77	12.43^∗∗^	0.20
	*SD*	0.16	0.16	0.17	0.10		


*^∗∗^*p* < 0.01.*

Children’s ratings of the importance of effort also differed across the three conditions (see **Table 1**) with a large effect size. *Post hoc* tests showed that children praised for effort (mean difference = 1.12, *SE* = 0.23, *p* < 0.01) and those not praised (mean difference = 0.71, *SE* = 0.24) rated effort as significantly more important than children praised for ability. No significant difference was found between children praised for effort and those not praised (mean difference = 0.42, *SE* = 0.24, *p* > 0.05). These results implied that children understood and were influenced by the experimenter’s type of praise.

### Effects of Praise on Children’s Failure Attribution

ANOVAs were conducted to compare children’s failure attributions in the three feedback conditions. The results (**Table 2**) showed that children differed in their attribution of low ability with a medium effect size. *Post hoc* tests indicated that children praised for ability were more likely to attribute failure to low ability than children praised for effort (mean difference = 0.51, *SE* = 0.25, *p* < 0.05) or not praised (mean difference = 0.70, *SE* = 0.25, *p* < 0.01). There was no difference between children praised for effort and those not praised (mean difference = 0.19, *SE* = 0.26, *p* > 0.05). There was also a significant main effect between groups in attributing failure to low effort with a large effect size. *Post hoc* tests showed that children praised for effort were more likely to attribute failure to low effort than the children praised for ability (mean difference = 0.89, *SE* = 0.25, *p* < 0.01) or not praised (mean difference = 0.88, *SE* = 0.26, *p* < 0.01). There was no significant difference between children praised for ability and those not praised in terms of low-effort attribution (mean difference = -0.02, *SE* = 0.26, *p* > 0.05). In terms of test anxiety, the main effect between the groups was significant with a large effect size. *Post hoc* tests showed that children praised for ability were more likely to attribute failure to test anxiety than children praised for effort (mean difference = 1.06, *SE* = 0.29, *p* < 0.01) and those not praised (mean difference = 1.81, *SE* = 0.30, *p* < 0.01). Children praised for effort were more likely to attribute failure to test anxiety than children not praised (mean difference = 0.75, *SE* = 0.30, *p* < 0.01). Finally, there was no noticeable difference in attributions to lack of time in the three feedback conditions.

**Table 2:** Descriptive statistics for children’s failure attribution.

		Ability group (*n* = 37)	Effort group (*n* = 35)	Control group (*n* = 31)	Total (*n* = 103)	*F*_(2,100)_	η*_p_*^2^
Low ability	*M*	2.54	2.03	1.84	2.16	4.23^∗^	0.08
	*SD*	1.24	0.98	0.82	1.07		
Low effort	*M*	3.08	3.97	3.10	3.39	8.02^∗∗^	0.14
	*SD*	0.89	1.07	1.22	1.13		
Lack of time	*M*	2.86	2.60	2.23	2.58	1.45	0.03
	*SD*	1.77	1.40	1.41	1.55		
Test anxiety	*M*	4.49	3.43	2.68	3.58	18.75^∗∗^	0.27
	*SD*	1.15	1.14	1.40	1.42		
*F*		16.60^∗∗^	18.87^∗∗^	7.52^∗∗^	29.93^∗∗∗^		
*df*		3,108	3,102	3,90	3,306		
η*_p_*^2^		0.32	0.36	0.20	0.23		


*^∗^*p* < 0.05; ^∗∗^*p* < 0.01; ^∗∗∗^*p* < 0.001.*

Repeated measures ANOVAs were also conducted to identify differences in failure attribution for the four different factors within the three groups (see **Table 2**). In the group praised for ability, the main effect of the attribution factor was significant with a large effect. *Post hoc* tests revealed that children praised for ability made much greater attributions to anxiety than to low ability (mean difference = 1.95, *SE* = 0.23, *p* < 0.01), low effort (mean difference = 1.41, *SE* = 0.21, *p* < 0.01), or lack of time (mean difference = 1.62, SE = 0.34, *p* < 0.01). In addition, children praised for ability attributed their performance more to low effort than to low ability (mean difference = 0.54, *SE* = 0.24, *p* < 0.05). In the group praised for effort, the main effect of the attribution factor was significant with a large effect. *Post hoc* tests showed that children praised for effort made much greater attributions to low effort than to low ability (mean difference = 1.94, *SE* = 0.26, *p* < 0.01), lack of time (mean difference = 1.37, *SE* = 0.32, *p* < 0.01), or test anxiety (mean difference = 0.54, *SE* = 0.25, *p* < 0.05). They also attributed their performance more to test anxiety than to low ability (mean difference = 1.40, *SE* = 0.25, *p* < 0.01) or lack of time (mean difference = 0.83, *SE* = 0.30, *p* < 0.05). In the control group, the main effect of the attribution factor was significant with a large effect. *Post hoc* tests indicated that children receiving no praise made much greater attributions to low effort than to low ability (mean difference = 1.26, *SE* = 0.21, *p* < 0.01) and lack of time (mean difference = 0.87, *SE* = 0.31, *p* < 0.01). They also attributed their performance more to test anxiety than to low ability (mean difference = 0.84, *SE* = 0.24, *p* < 0.01).

### Effects of Praise on Children’s Self-Handicapping

Claimed self-handicapping was indexed by the children’s level of test anxiety. As shown in **Table 3**, a one-way ANOVA identified significant variance in children’s test anxiety with a large effect size. *Post hoc* tests showed that children praised for ability reported significantly more anxiety than the children praised for effort (mean difference = 0.55, *SE* = 0.24, *p* < 0.05) and those not praised (mean difference = 1.44, *SE* = 0.25, *p* < 0.01). While, appropriate caution should be used when drawing a conclusion with the statistical results. Because there is overlapping confidence interval on the test anxiety between the ability group (95% CI = [3.59, 4.25]) and effort group (95% CI = [3.03, 3.72]). Children praised for effort also claimed significantly more anxiety than those not praised (mean difference = 0.89, *SE* = 0.25, *p* < 0.05). Children’s behavioral self-handicapping was indexed by time spent working on the third set of matrices. Controlling the time spent working on set 1, covariance analysis found significant variance among the three groups in time spent working on set 3 with a large effect size. *Post hoc* tests showed that children praised for ability spent less time solving problems after failure than children praised for effort (mean difference = -0.84, *SE* = 0.17, *p* < 0.01) and those not praised (mean difference = -0.88, *SE* = 0.18, *p* < 0.01). No significant difference was found between children praised for effort and those not praised (mean difference = -0.04, *SE* = 0.18, *p* > 0.05; **Table 3**).

**Table 3:** Descriptive statistics of claimed and behavioral self-handicapping among children.

		Ability group (*n* = 37)	Effort group (*n* = 35)	Control group (*n* = 31)	Total (*n* = 103)	*F (df)*	η*_p_*^2^
Test anxiety	*M*	3.92	3.37	2.48	3.26	16.72^∗∗^	0.25
	*SD*	0.17	0.17	0.18	0.10	(2,100)	
Time of set 1	*M*	4.11	4.03	3.77	3.98	2.19	0.04
	*SD*	0.12	0.12	0.13	0.07	(2,100)	
Time of set 3	*M*	2.98	3.82	3.86	3.55	16.40^∗∗^	0.25
	*SD*	0.12	0.12	0.13	0.07	(3,99)	


*^∗∗^*p* < 0.01.*

### Effects of Praise on Children’s Performance

One-way ANOVA revealed significant variance in performance change from the first to third problem set among the three groups, *F*_(2,100)_ = 7.17, *p* < 0.01, η*_p_*^2^ = 0.13, with a medium effect size. *Post hoc* tests indicated that children praised for ability made significantly less improvement than children praised for effort (mean difference = -0.92, *SE* = 0.30, *p* < 0.01) and those not praised (mean difference = -1.02, *SE* = 0.30, *p* < 0.01). There was no significant difference between children praised for effort and those not praised (mean difference = -0.10, *SE* = 0.31, *p* > 0.05; **Table 4**).

**Table 4:** Descriptive statistics for children’s performance.

		Ability group (*n* = 37)	Effort group (*n* = 35)	Control group (*n* = 31)	Total (*n* = 103)	*F* (2,100)	η*_p_*^2^
P1	*M*	7.22	6.51	6.94	3.26	2.32	0.04
	*SD*	1.36	1.42	1.39	0.10		
P3	*M*	7.32	7.54	8.06	3.98	3.35^∗^	0.06
	*SD*	1.25	1.15	1.18	0.07		
Performance change	*M*	0.11	1.03	1.13	3.55	7.17^∗∗^	0.25
	*SD*	1.15	1.27	1.34	0.07		


*P1, performance on the first set of problems; P3, performance on the third set of problems. ^∗^*p* < 0.05.*

## Discussion

The purpose of this study was to investigate the absolute effects of ability and effort praise on children’s failure attributions, self-handicapping, and subsequent post-failure performance. The results showed that the children praised for ability after good performance are more inclined to place higher relative importance on ability for their performance after subsequent failure compared to the children praised for effort or those not praised. Previous findings from Western educational settings also demonstrated that praising a child’s ability for their good performance made them more inclined to attribute future failure to lack of ability (Mueller and Dweck, 1998). In addition, our study extended the established understanding of these effects by comparing four failure attribution factors within each group. Compared to the children who received effort praise and simple informational feedback, the children praised for ability were more likely to attribute their failure to non-ability factors, such as test anxiety, which was consistent with our predictions. The self-worth theory of achievement motivation argues that individuals will strive to avoid failure or to alter its meaning by using defensive attributional strategies as a means of self-protection (Martin et al., 2003). In the current study, we also demonstrated that compared with effort praise and simple informational feedback, ability praise leads children to adopt claimed self-handicapping by reporting higher levels of test anxiety as well as behavioral self-handicapping by spending less time on tasks when they faced subsequent failure. Usually, uncertainty about one’s ability or potential threats to one’s self-esteem is the driving force for self-handicapping (Covington, 1992). Children who are praised for ability will adopt more fixed ability beliefs and consider the subsequent failure as evidence of lacking ability and threats on self-worth (Amemiya and Wang, 2018). So, they will create self-handicaps. Self-handicapping is grounded in attribution theory, whereby the certainty with which a cause can be attributed to success or failure depends on the number of alternative possible factors (Weiner, 1994). Covington (1992, 2009) argued that individuals were not most afraid of failure, but of their failure being attributed to their low ability. The presence of self-handicaps provides individuals the chance to transfer attribution for poor performance from low ability to the prepared handicaps when they have to face failure (Berglas and Jones, 1978). Unlike claimed self-handicapping, behavioral handicaps, which create an actual obstacle that can directly undermine subsequent task performance, are costlier (Leary and Shepperd, 1986; McCrea et al., 2008; Schwinger et al., 2014). Therefore, it was not surprising that the children who received praise for ability demonstrated only slight improvements in scores over time, even though they had been familiar with the format of the tasks in this study. This contrasted with the more striking improvements in post-failure scores achieved by the children in both the effort praise and no-praise conditions, who seemed to associate effort with good performance and applied themselves accordingly. Mueller and Dweck (1998) found that children praised for ability may display a helpless response pattern after encountering setbacks, including less persistence and impaired performance. These findings are also in line with previous research on children’s self-handicapping, which indicated that behavioral self-handicapping undermines performance (Snyder et al., 2014).

The most striking finding was that the children praised for effort were also more likely to report higher levels of text anxiety and contribute their failure to text anxiety than those in the control condition, and report the same levels of text anxiety as those praised for ability. These results suggest that children praised for effort use the claimed self-handicapping and defensive attributional strategies more than children who only receive simple informational feedback. Although Chinese people tend to emphasize the importance of effort (Lewis, 1995; Salili, 1996), our study found that the benefits of praise for effort were limited if hard work still resulted in a setback, and simple informational feedback was the best response in this case. One possible explanation of this result is that older children (at ages older than 11) tend to believe that effort and ability have a compensatory relationship, or that effort and ability are inversely related (Nicholls, 1978; Henderlong and Lepper, 2002). It is clear that in some conditions, effort praise may be damaging because it conveys a message of low ability. For example, Lam et al. (2008) found that the effects of effort praise on children’s motivation were moderated by the children’s beliefs in the inverse relationship between effort and ability, the motivational effects of effort praise depend on children’s beliefs in the ability–effort relationship. Effort praises are commonly considered as adults’ low expectations about their abilities by adolescents and consequently reduce their motivation to learn and overcome setbacks (Amemiya and Wang, 2018). Our study found no significant difference in post-failure achievement between the children praised for effort and the children who were not praised. One possible explanation for this finding is that effort praise also made the children anticipate threats to their post-failure achievement. Fortunately, they only used claimed self-handicapping in the process of self-protection and did not use behavioral self-handicapping, which is much costlier. Claimed handicaps, such as reports of test anxiety, can serve as an excuse for failure but do not necessarily decrease one’s chances of being successful as behavioral handicaps do (Schwinger et al., 2014).

Furthermore, our study found that children who received informational feedback without praise for good performance were more inclined to rate effort as more significant than ability for their performance and attribute their subsequent failure to a lack of effort, and they were able to achieve the same improvement in the post-failure task as children praised for effort. A culture-specific explanation for the present Chinese sample is that within collectivistic cultures, people’s beliefs about achievement outcomes tend to center on hard work (Lewis, 1995). Indeed, cross-cultural studies have found that mothers and their children in China and Japan place more importance on effort over ability in explaining their achievement outcomes, while the opposite is the case among Americans (Stevenson et al., 1990). It was surprising that simple informational feedback had the same positive effects as praise for effort, indicating that children could be motivated by their own good performance. It is very important for teachers and parents to provide their children some informational feedback about their academic achievement and behavior performance, but this does not imply that we should give children a large number of positive evaluative responses. These findings were consistent with previous research. For example, Skipper and Douglas (2012) also found that objective feedback may be sufficient to encourage the growth mindset, teachers may not necessarily need to go out of their way to provide evaluative comments on a learner’s performance.

### Limitations, Future Directions, and Practical Implications

This is the first study that investigated the effects of praise for ability and effort in the cultural context of Mainland China, and the research paradigm included a no-praise group, which allowed us to investigate the absolute effects of praise. In addition, we obtained some novel findings and identified the limited effects of effort praise. Despite its contributions and novel findings, this study also had several limitations. First, some of the participants in this study were familiar with the standard progressive matrices from their Mathematical Olympiad training classes, so they did not find these problems very challenging, which probably influenced the effects of praise on those children. Second, we used a modest-sized sample of children from Beijing, which was not fully representative of the cultural context of Mainland China, and also influenced the stability of the results, such as the striking improvement in post-failure scores achieved by children in control group, future research with bigger sample sizes will yield more stable results. The third limitation is that only using one item to measure claimed and behavioral self-handicapping without considering other indexes may reduce the reliability of our findings. Finally, the current study took the Raven’s progressive matrices, a psychometric test of general ability without any curricular relevance, as experimental materials, which may limit the practical implications. To date, few studies have used curricular relevant tasks to examine the effects of praise, such as math tasks (Weaver and Watson, 2004) and English crossword puzzle tests (Leis, 2017). We could take curricular tasks (e.g., mathematical problems, reading comprehension, or written composition) to improve the ecological validity of the study in the future research.

Given the importance of praise for children’s motivation and behaviors, further studies are needed to explore the positive or disruptive effects of praise on child development. Our study replicated Mueller and Dweck’s (1998) study with the inclusion of a no-praise control group to explore the absolute effects of ability versus effort praise on children’s subsequent failure attribution and self-handicapping. It would be interesting to replicate this study in diverse cultural settings to verify the results with larger sample sizes. Furthermore, Chinese individuals are more inclined to attribute their achievement outcomes to effort. More studies need to be conducted to determine the conditions in which praise for effort has or does not have positive effects on children’s cognition, motivation, and achievement. In addition, we suggest that other forms of praise and their effects be included in future research. For example, it would be worth conducting an examination of how praise affects children’s motivations in light of social comparison, and whether public praise—such as that given by a teacher in front of a classroom full of students—has different and beneficial effects on performance, self-appraisal, and broader development. Finally, the experimental tasks could be presented online to make a better control of the quality of the dart in future study.

In China, the conventional wisdom holds that praise is harmful for child development as it may make children feel complacent. In the 1990s, education reform with an emphasis on positive evaluation was carried out, so that praise, especially praise for ability, now prevails in Chinese homes and schools. Teachers and parents extensively use ability and effort praise to improve children’s motivation and performance. Hence, educators and researchers are seeking to explore the differential effects of these types of praise on children’s cognitions, emotions, and behaviors. The results of this study may prompt educators to rethink the educational reforms implemented in families and schools that excessively emphasize the importance of positive evaluative feedback on children’s development. These findings also have significant implications for parenting and school education. They can assist parents and educators in taking advantage of simple informational feedback to benefit child development and caution parents and teachers that we should administer ability praise with caution. Effort praise should not haphazardly use for older children (at ages older than 11) because of their beliefs in the inverse relationship between ability and effort.

## Conclusion

In conclusion, the present research explored the differential effects of ability and effort praise in an attempt to account for the etiology of self-serving attribution and self-handicapping behaviors, and made a unique contribution to the previous literature basing on the self-worth theory. The results showed that ability praise led children to use self-serving failure attribution as well as claimed and behavioral self-handicapping and to achieve less improvement on post-failure tests. Strikingly, effort praise also had some disruptive effects for older children (at ages older than 11) —children praised for effort reported higher levels of text anxiety and they were more likely to then attribute their failure to text anxiety compared with those who only received simple informational feedback.

## Ethics Statement

This study was carried out in accordance with the recommendations of the Research Ethics Committee of Capital Normal University. All of the subjects gave written informed consent in accordance with the Declaration of Helsinki. The protocol was approved by the Research Ethics Committee of Capital Normal University.

## Author Contributions

SX and XL conceived and designed the study. XG, YJ, and MA collected and analyzed the data. SX, XL, XG, YJ, and MA completed and modified the manuscript. MA provided language editing for the manuscript.

## Conflict of Interest Statement

The authors declare that the research was conducted in the absence of any commercial or financial relationships that could be construed as a potential conflict of interest.
